# A Synopsis of the Medical Botany of Illinois

**Published:** 1884-09

**Authors:** I. M. C. Carter


ARTICLE II.

### A SYNOPSIS OF THE MEDICAL BOTANY OF ILLINOIS. *By* I. M. C. CARTER, M.A., M.D., PH.B.

(*Continued from August Number.*)

Order Cistaceæ (Rock-rose Family). 2 genera, 2 species:

Genus Helianthemum (Rock-rose). H. Canadense (Frost-weed). Medical properties: Aromatic, alterative, tonic, astringent, slightly bitter.

Genus Lechea (Pinweeds). L. major (Pinweed). Medical properties: Tonic, antipyretic, antiperiodic.

Order Drosceraceæ (Sundew Family). 1 genus, 2 species:

Genus Drosera (Sundew). D. longifolia (Long-leaved Sundew); D. rotundifolia (Round-leaved Sundew). Medical properties: Acrid, detergent, rubefacient, pectoral, aphrodisiac.

Order Hypericaceæ (St. John's-wort Family). 2 genera, 4 species:

Genus Hypericum (St. John's-wort). *H. perforatum*, or H. officinale (Common St. J.); H. mutilum (Low Century); H. sarothra (Nitweed, Pinweed, Orange-grass). Medical properties: Bitter, aromatic, astringent, tonic, resolvent, nervine.

Genus Elodes (Marsh St. John's-wort). E. Virginica. Medical properties: Same as preceding genus.

Order Caryophyllaceæ (Pink Family). 7 genera, 8 species:

Genus Lychins (Lychins Cockle). *L. githago* (Corn Cockle). Medical properties: Seed—acrid, depurative, purgative; root—astringent, vulnerary.

Genus Silene (Catchfly Campion). *S. inflata* (Bladder Campion); S. Virginica (Fire Pink Catchfly). Medical properties: Nervine, vermifuge (like Spigelia).

Genus Saponaria (Soapwort). *S. officinalis* (Bouncing Bet,

Soapwort, Benisewort). Medical properties: Tonic, diaphor etic, alterative, antisyphilitic.

Genus Vaccaria (Cow-herb). *V. vulgaris* (Field Soapwort). Medical properties: Seed—diuretic; plant—galactagogue.

Genus Cerastium (Mouse-ear, Chickweed). *C. vulgatum.* Medical properties: Refrigerant, nutrient.

Genus Stillaria (Startwort, Chickweed). *S. media* (Common Chickweed). Medical properties: Alterative, mucilaginous, demulcent, refrigerent, discutient, detergent.

Genus Mollugo (Indian Chickweed). M. verticillata (Carpet weed). Medical properties: Refrigerant, diuretic.

Order Portulacaceæ (Purslane Family). 1 genus, 1 species:

Genus Portulaca (Purslane). *P. oleracea* (Common Purslane). Medical properties: Antiseptic, diuretic, aperient, vulnerary. Also called Pursley.

Order Malvaceæ (Mallow Family). 4 genera, 7 species:

Genus Malva (Mallow). *M. rotundifolia* (Round-leaved M.); *M. sylvestris vel M. vulgaris* (High M.). Medical properties: Mucilaginous, diuretic, emollient, pectoral.

Genus Sida. *S. spinosa.* Medical properties: Diuretic, demulcent.

Genus Abutilon (Indian Mallow). *A. avicennæ* (Velvet Leaf). Medical properties: Demulcent, mucilaginous, diuretic.

Genus Hibiscus (Rose Mallow). H. moscheutos (Musk Seed, Swamp Rose M.); *H. trionum* (Bladder Keturia); *H. esculentus.* (Gumbo, Okra). Medical properties: Mucilaginous, demulcent.

Order Tiliaceæ (Linden Family). 1 genus, 1 species:

Genus Tilia (Linden Rosewood). T. Americana (Basswood). Medical properties: Bark—emollient, demulcent, mucilaginous; flowers—cephalic, stimulant, sedative. Also called Linn, Linden, and Lime-tree.

Order Linaceæ (Flax Family). 1 genus, 2 species:

Genus Linum (Flax). *L. usitatissimum* (Common Flax). L. Virginanum (Wild Flax). Medical properties: Demulcent, mucilaginous, emollient.

Order Geraniaceæ (Geranium Family). 2 genera, 4 species:

Genus Oxalis (Wood Sorel). O. stricta (Yellow Wood S.); O. Violacea (Violet Wood S.). Medical properties: Acid, refrigerant, diuretic, antiscorbutic, irritant.

Genus Geranium (Cranesbill). G. maculatum (Wild Cranesbill, Spotted C.); G. Carolinianum (Carolina Geranium). Medical properties: Astringent, styptic, antiseptic, bitter, tonic, febrifuge, nephritic.

Order Rutaceæ (Rue Family). 2 genera, 1 species:

Genus Xanthoxylum (Prickly Ash). Z. Americanum (Northern Prickly Ash). Medical properties: Stimulant, sialagogue, alterative, tonic.

Genus Ptelea (Hop tree, Shrubby Trefoil). P. trifoliata (Wafer Ash, Hop tree, Swamp Dogwood). Medical properties: Same as preceding.

Order Anacardiaceæ (Cashew Family). 2 genera, 9 species:

Genus Rhus (Sumach). *R. cotinus* (Smoke tree, Venetian Sumach); R. toxicodendron (Poison Ivy, Poison Oak): R. radicans; R. venenata (Poison Sumach, Poison Dogwood); R. typhina (Staghorn S.); R. glabra (Smooth S.); R. copallina (Dwarf S.); R. aromatica (Fragrant S.). Medical properties: Tonic, astringent, antiseptic; sometimes rubefacient, vesicant.

Genus Ailanthus. *A. glandulosus* (Chinese Sumach, Tree of Heaven). Medical properties: Nervine, sedative; bark—vermifuge.

Order Vitaceæ (Vine Family). 2 genera, 4 species:

Genus Vitis (Grape). V. æstivalis (Summer Grape); V. cordifolia (Winter, or Frost Grape); V. vulpina (Muscadine, Southern Fox Grape). Medical properties: Acid, refrigerant, antiscorbutic, nutrient.

Genus Ampelopsis (Virginia Creeper). A. quinquefolia (American Ivy). Medical properties: Alterative, astringent, tonic, expectorant.

Order Rhamnaceæ (Buckthorn Family). 2 genera, 3 species:

Genus Ceanothus (Red Root, New Jersey Tea). C. Americanus (New Jersey Tea); C. ovalis. Medical properties: Astringent, expectorant, sedative, antisyphilitic, stimulant. Similar in its effects to lobelia.

Genus Frangula (Buckthorn, Alder). F. Caroliniana (Alder, Buckthorn). Medical properties: Astringent, cathartic, anthelmintic. This species is sometimes called Rhamnus frangula.

Order Celastraceæ (Staff Tree Family). 2 genera, 3 species:

Genus Celastus (Shrubby Bitter-sweet, Staff Tree). C. scandens (Wax-work, Climbing Bitter-sweet). Medical properties: Bark of root—alterative, diuretic, hepatic, narcotic, stimulant, antisyphilitic.

Genus Euonymous (Spindle Tree). E. atropurpureus (Wahoo, Burning Bush); E. Americanus (Strawberry Bush). Medical properties: Tonic, alterative, diuretic, laxative, expectorant, antiperiodic; seed—cathartic.

Order Sapindaceæ (Soap-berry Family). 3 genera, 6 species:

Genus Æsculus (Horse Chestnut, Buckeye). Æ. glabra (Fœtid, or Ohio Buckeye). Medical properties: Tonic, febrifuge, narcotic.

Genus Acer (Maple). *A. pseudo-palatus* (Sycamore Maple); A. saccharinum (Rock or Sugar Maple); A. nigrum (Black M. or Sugar Tree); A. rubrum (Red or Swamp M.). Medical properties: Astringent, bitter, tonic, antiperiodic.

Genus Negundo (Ash-leaved Maple, Box Elder). N. aceroides. Medical properties: Similar to preceding genus.

Order Polygalaceæ (Polygala Family). 1 genus, 5 species:

Genus Polygala (Milkwort). P. incarnata; P. sanguinea; P. senega (Seneca Snakeroot); P. polygama (Bitter Polygala); P. paucifolia (Fringed Polygala). Medical properties: Bitter, stimulant, sialagogue, expectorant, diuretic, tonic, diaphoretic, emmenagogue.

Order Leguminosæ (Pulse Family). 14 genera, 21 species:

Genus Lupinus (Lupine). L. perennis (Wild Lupine). Medical properties: Bitter, anthelmintic, emollient (used as cataplasm).

Genus Melilotus (Melilot, Sweet Clover) *M. alba or M. officinalis* (White Melilot, Bokhara or Tree Clover). Medical properties: Aromatic, pectoral, expectorant, diuretic, emollient, discutient.

Genus Trifolium (Clover, Trefoil). T. arvense (Rabbit-foot, Stone Clover), *T. pratense* (Red Clover); T. repens (White Clover). Medical properties: Pectoral, antidysenteric, detergent, depurent, alterative, laxative. Red clover is somewhat noted as a *cancer remedy*.

Genus Amorpha (False Indigo). A. fructicosa; A. canescens (Lead Plant). Medical properties: Alterative.

Genus Psoralea. P. melilotoides. Medical properties: Stim ulant, tonic.

Genus Stylosanthes (Pencil Flower). S. elatior. Medical properties: Uterine sedative in gestation, tonic in parturition.

Genus Lespedeza (Bush Clover). L. violacea, var. sessiliflora. Alterative, diuretic.

Genus Tephrosia (Hoary Pea). T. Virginiana (Goat's Rice, Catgut). Medical properties: Stimulant, tonic, antisyphilitic, vermifuge.

Genus Robinia (Locust Tree). R. pseudacacia (False Acacia, Common Locust). Medical properties: Bark of root—emetic, cathartic, expectorant, tonic; flowers—anti-spasmodic.

Genus Lathyrus (Vetchling). L. maritimus (Beach Pea). Medical properties: Emollient; used in poultices,

Genus Baptisia (False Indigo). B. tinctoria (Wild I.); B. leucantha; B. australis (Blue False I.). Medical properties: Alterative, antiseptic, emetic, cathartic, emmenagogue, discutient.

Genus Cercis (Judas Tree, Red Bud). C. Canadensis (American R.). Medical properties: Antiscorbutic. Fruit edible.

Genus Cassia (Senna). C. marilandica (Wild Senna); C. chamæcrista (Large-flowered Sensitive or Partridge Pea); C. nictitans (Wild Sensitive Plant). Medical properties: Cathartic, anthelmintic, diuretic, antisyphilitic.

Genus Gymnocladus (Kentucky Coffee Tree). G. Canadensis). Medical properties: Seeds and pods—emetic, nervine; leaves—cathartic.

Order Rosaceæ (Rose Family). 11 genera, 35 species.

Genus Prunus (Almond, Peach, Plum, etc.). P. Pennsylvanica (Wild Red Cherry); P. serotina (Wild Black C.); P. Virginiana (Choke C.). Medical properties: Bark—bitter, tonic, antiperiodic, astringent, expectorant, stimulant, nervine, sedative.

Genus Spiræa (Meadow Sweet). S. opulifolia (Nine Bark); S. tomentosa (Hardhack, Steeple Bush); S. lobata (Queen of the Prairie). Medical properties: Astringent, tonic.

Genus Gillenia (Indian physic). G. stipulacea (Bowma*n*'s Root, American Ipecac). Medical properties: Emetic, cathartis, sudorific, expectorant, tonic.

Genus Geum (Avens). G. vernum (Early Water A.); G. rivale (Water A., Purple A., Chocolate Root); G. strictum (Upright A., Field A., Herb Bennet); G. Virginianum (White A.). Medical properties: Astringent, tonic, sedative, antipyretic, stomachic. Sometimes used as a substitute for tea and coffee.

Genus Potentilla (Cinque-foil, Five-finger). P. Norvegica (Norway Cinque-foil); P. Canadensis (Five-finger); P. anserina (Silver Weed); P. Fructicosa (Shrubby Cinque-foil); P. palustris (Marsh Five-finger). Medical properties: Astringent, tonic.

Genus Fragaria (Strawberry). F. vesca (Common S.); F. Virginiana (Wild Strawberry). Medical properties: Fruit—antiscorbutic, refrigerant; leaves—astringent; both diuretic.

Genus Rubus (Bramble). *R. idæus* (Garden Raspberry); R. strigosus (Wild Red R.); R. occidentalis (Wild Black R., Thimbleberry, Black Cap); R. villosus, (High or Common Blackberry); R. Canadensis (Low B., Dewberry). Medical properties: Astringent, diuretic, laxative, antiscorbutic, refrigerant; bark of the root—antiemetic.

Genus Agrimonia (Agrimony). A. Eupatoria (Common A.); A. parviflora (Small-flowered A.). Medical properties: Mild astringent, stomachic.

Genus Rosa (Rose). R. Carolina (Swamp R.); R. lucida (Dwarf Wild R.); R. blanda (Early Wild R.). Medical properties: Astringent, tonic.

Genus Cratægus (Hawthorn, White Thorn). C. coccinea (Scarlet-fruited T.); C. tomentosa (Black or Pear T.); C. punctata (Red Haw); C. crus-galli (Cockspur T.). Medical properties: Astringent, tonic, expectorant. Bark, leaves and fruit used.

Genus Pyrus (Pear, Apple). P. coronaria (Crab Apple); P. arbutifolia (Chokeberry); P. Americana (American Mountain Ash). Medical properties: Astringent, tonic, detergent, antiscorbutic, esculent.

Order Saxifragaceæ (Saxifrage Family). 5 genera, 6 species:

Genus Ribes (Currant, Gooseberry). R. floridum (Wild Black Currant). Medical properties: Fruit—diuretic, diaphoretic, anti-scorbutic; bark—lithontryptic.

Genus Hydrangea. H. arborescens (Wild H., Seven-barks). Medical properties: Root—diuretic, lythontryptic; leaves—tonic, sialagogue, diuretic, cathartic.

Genus Parnassia (Grass of Parnassus). P. Caroliniana. Medical properties: Seed—diuretic, aperient, sedative (locally).

Genus Heuchera (Alum Root). H. Americana (Common A.). Medical properties: Root—powerfully astringent.

Genus Mitella (Mitre-wort, Bishop's-cap). M. nuda (Coolwort); M. dyphylla. Medical properties: Astringent, diuretic.

Order Hamamelaceæ (Witch Hazel Family). 2 genera, 2 species:

Genus Hamamelis (Witch Hazel). H. Virginiana. Medical properties: Bark and leaves—astringent, tonic, sedative, discutient.

Genus Liquidambar (Sweet Gum Tree, Bilsted). L. styraciflua. Medical properties: Bark—nervine; flowers—aromatic.

Order Halorageæ (Water Milfoil Family). 1 genus, 1 species:

Genus Hippuris (Mare's Tail). H. vulgaris. Medical properties: Diuretic, astringent.

Order Onagraceæ (Evening Primrose Family). 4 genera, 7 species:

Genus Circæa (Enchanter's Nightshade). C. lutetiana. Medical properties: Resolvent, vulnerary.

Genus Epilobium (Willow Herb). E. palustre; E. augustifolium (Fire-weed, Willow-herb); E. coloratum. Medical properties: Tonic, astringent, demulcent, emollient.

Genus Œnothera (Evening Primrose). Œ. biennis (Common Evening P.). Medical properties: Emollient, astringent.

Genus Ludwigia (False Loose Strife). L. alternifolia (Seedbox); L. palustris (Phthisic Weed, Water Purslane). Medical properties: Pectoral, expectorant. Used in asthma.

Order Lythraceæ (Loose Strife Family). 1 genus, 1 species:

Genus Lythrum (Loose Strife). L. alatum. Medical properties: Astringent, mucilaginous, antisyphilitic.

Order Cactaceæ (Cactus Family). 1 genus, 1 species:

Genus Opuntia (Prickly Pear, Indian Fig). O. vulgaris (Common Prickly Pear). Medical properties: Refrigerant.

Order Cucurbitaceæ (Gourd Family). 1 genus, 1 species:

Genus Sicyos (Star Cucumber). S. angulatus. Medical properties: Seed—diuretic, purgative.

Order Umbelliferæ (Parsley Family). 7 genera, 11 species:

Genus Archangelica. A. atropurpurea (Great Angelica, Purple A.); A. hirsuta. Medical properties: Root and seed—cathartic, stimulant, emmenagogue.

Genus Heracleum (Cow Parsnip). H. Lanatum. Medical properties: Stimulant, antispasmodic, carminative, narcotic.

Genus Pastinaca (Parsnip). *P. sativa* (Common Parsnip). Medical properties: Seed and tops—diuretic.

Genus Æthusa (Fool's Parsley). *Æ. cynapium.* Medical properties: Emetic, irritant; poisonous.

Genus Thaspium (Meadow parsnip). T. barbinode; T. aureum; T. trifoliatum; T. atropurpureum. Medical properties: Alterative, diaphoretic, antisyphilitic.

Genus Bupleurum (Thorough Wax). *B. rotundifolium.* Medical properties: Aromatic.

Genus Discoplura (Mock Bishop Weed). D. capillacea var-costata. Medical properties: Aromatic, pungent, tonic, diuretic, carminative.

Order Araliacæ (Ginseng Family). 1 genus, 4 species:

Genus Aralia. A. spinosa (Angelica Tree, Hercules' Club); A. racemosa (Spikenard); A. nudicaulis (Wild Sarsaparilla, American S.); A. quinquefolia (Ginseng). Medical properties: Aromatic, alterative, pectoral, diaphoretic, depurative, stimulant, balsamic, sialagogue, sudorific, emetic.

Genus Conium (Poison Hemlock). *C. Maculatum.* Medical properties: Narcotic, sedative, anodyne, anti-spasmodic, diuretic,deobstruent.

Order Cornaceæ (Dogwood Family). 2 genera, 7 species:

Genus Cornus (Cornel, Dogwood). C. Canadensis (Dwarf Cornel, Bunchberry); C. florida (Flowering Dogwood); C. sericea (Silky D., Kinnikinik); C. circinata (Round-leaved D., Green Osier); C. paniculata (Panicled Cornel, White Cornel); C. alternifolia (Alternate-leaved C.). Medical properties: Bitter, tonic, astringent, diaphoretic, antiperiodic.

Genus Nyssa (Tupelo, Pepperidge, Sour Gum). N. multiflora (Black Gum, Common T.). Medical properties: Acid, refrigerant.

Order Caprifoliaceæ (Honeysuckle Family). 6 genera, 13 species:

Genus Linnæa (Twin-flower). L. borealis. Medical properties: Bitter, subastringent, antirheumatic.

Genus Triosteum (Feverwort, Horse Gentian). T. perfoliatum; T. augustifolium. Medical properties: Emetic, tonic, diaphoretic, laxative.

Genus Symphoricarpus (Snowberry). S. racemosus (Snowberry); S. occidentalis (Wolfberry); S. vulgaris (Indian Currant). Medical properties: Root—Astringent, alterative, tonic.

Genus Diervilla (Bush Honeysuckle). D. trifida. Medical properties: Diuretic, astringent, alterative.

Genus Viburnum (Arrow Wood). V. lentago (Sheepberry); V. prunifolium (Black Haw, Sweet H.); V. dentatum (Arrow Wood); V. acerifolium (Dock Mackie); V. opulus (Cranberry Tree). Medical properties: Bark—diuretic, detergent, tonic, astringent, antiperiodic, antispasmodic, expectorant, alterative; bark of root of Black Haw—a valuable uterine sedative.

Genus Sambucus (Elder). S. Canadensis (Common Elder).

Medical properties: Bark—cathartic, emetic; flowers—diuretic, diaphoretic; exanthematous.

Order Rubiaceæ (Madder Family). 3 genera, 7 species:

Genus Galium (Bedstraw, Cleavers). G. asprellum (Rough Bedstraw); G. trifidum (Small B.); G. triflorum (Sweet-scented B.); G. aparine (Cleavers, Goosegrass); G. circæzans (Wild Liquorice). Medical properties: Diuretic, diaphoretic, demulcent, expectorant; herb of Goosegrass—aperient, refrigerant.

Genus Mitchella. M. repens (Partridge Berry). Medical properties; Diuretic, astringent, alterative, tonic, parturient.

Genus Cephalanthus. C. occidentalis (Button-bush). Medical properties: Tonic, laxative, diuretic, aperient, febrifuge.

Order Valerianaceæ (Valerian Family.) 1 genus, 2 species:

Genus Valeriana (Valerian). V. edulis (Oregon Tobacco); V. pauciflora (Wild Valerian). Medical properties: Aromatic, stimulant, tonic, anodyne, nervine, antispasmodic; the former, esculent.

Order Dipsaceæ (Teasel Family). 1 genus, 1 species:

Genus Dipsacus (Teasel). *D. sylvestris* (Wild T.). Medical properties: Root—diuretic, sudorific, stomachic.

Order Compositæ (Composite Family). 43 genera, 90 species:

Genus Cirsium (Thistle). *C. arvense* (Canada T.). Medical properties: Tonic, diuretic, hepatic, antibilious.

Genus Lappa (Burdock). *L. officinalis.* Medical properties: Root—alterative, diuretic, depurative; seed—diuretic, alterative.

Genus Centamæ (Star Thistle). *C. cyanus* (Bluebottle). Medical properties: Stomachic, tonic.

Genus Xanthium (Cocklebur, Clotbur). *X. strumarium* (Common C.); *X. spinosum* (Spiney C.) Medical properties: Same as Lappa officinalis.

Genus Ambrosia (Ragweed). A. trifida (Great Ragweed);

A. artemisæfolia (Romanweed, Hogweed, Bitterweed), Medical properties: Astringent, stimulant, detergent, antiseptic.

Genus Matricaria (Wild Chamomile). M. discorida. Medical properties: Tonic, stomachic, carminative.

Genus Tanacetum (Tansy). *T. Vulgare* (Common Tansy.) Medical properties: Aromatic, tonic, emmenagogue, diaphoretic.

Genus Artemisia (Wormwood). *A. absinthium* (Wormwood); *A. vulgaris* (Mugwort); A. ludoviciana (Western M.); A. biennis (Biennial Wormwood); A. abrotanum (Southernwood); A. Canadensis (Canada W.). Medical properties: Bitter, tonic, deobstruent, astringent, anthelmintic, diaphoretic.

Genus Erechthites (Fireweed). E. hieracifolia (F.) Medical properties: Emetic, tonic, astringent, alterative.

Genus Gnaphalium (Everlasting, Cudweed). G. polycephalum (Sweet-scented E.); G. uliginosum (Low Cudweed, Mouse Ear). Medical properties: Astringent, diaphoretic, pectoral, sudorific, stomachic, mucilaginous, diuretic; juice of former, anaphrodisiac.

Genus Antennaria (Everlasting Immortelle). A. dioricum (Life Everlasting); A. margaritæa (Pearl-flowered E.); A. plantaginifolia (Plantain-leaved E., White Plantain). Medical properties: Astringent, styptic, anodyne, vermifuge, pectoral, diuretic.

Genus Vernonia (Ironweed). V. Noveboracensis (Common Ironweed); V. fasciculata. Medical properties: Bitter, tonic, deobstruent, alterative, depurative.

Genus Liatris (Button Snakeroot, Blazing Star). L. squarrosa (Common Blazing S.); L. cylindracea; L. Scariosa (Rattlesnake's Master); L. spreata (Button Snakeroot). Medical properties: Diuretic, stimulant, tonic, diaphoretic, emmenagogue, alterative.

Genus Eupatorium (Thoroughwort, Boneset). E. purpureum (Purple Bonset or Thoroughwort, Trumpet-weed, Joe-Pye weed, Queen-of-the-Meadow); E. Purfoliatum (Boneset, Thoroughwort); E. sessifolium (Upland Boneset); E. ageratoides (White Lanicle, White Snake-root); E. Aromaticum. Medical properties: Tonic, aromatic, bitter, astringent, diuretic, diaphoretic, antispasmodic, expectorant, aperient, emetic, anti-periodic.

Genus Kuhnia (False Boneset). K. Eupatorioides. Medical properties: Bitter, tonic, diaphoretic.

Genus Conoclinum (Mist Flower). C. coelestinum. Medical properties: Diaphoretic, antispasmodic, expectorant.

Genus Cacalia (Indian Plantain). C. suraveolus; C. reniformis (Great Indian Plantain); C. atriplicifolia (Pale Indian Plantain); C. tuberosa (Tuberous Indian P.). Medical properties: Emollient, pectoral, like Marsh Mallow.

Genus Tussilago (Colt's-foot). T. farfara. Medical properties: Demulcent, emollient, tonic, errhine, expectorant, pectoral.

Genus Senecio (Groundsel). S. aureus (Golden Ragwort, Squaw-weed); S. Colbatus (Butterweed). Medical properties: Astringent, tonic, diuretic, diaphoretic, emmenagogue, expectorant.

Genus Inula (Elecampane). *I. helenium.* Medical properties: Expectorant, emollient, tonic, diuretic, diaphoretic.

Genus Solidogo (Golden-rod). S. bicolor; S. virga-aurea; S. rigida; S. odora (Sweet Golden-rod); S. gigantia. Medical properties: Carminative, stimulant, diaphoretic, diuretic, astringent, styptic.

Genus Aster (Aster, Starwort). A. undulatus; A. cordifolius; A. aestivus (Sampson-Snakeroot); A. puniceus (Cohosh). Medical properties: Antispasmodic, alterative, antirheumatic, aromatic, tonic, nervine, diuretic.

Genus Erigeron (Fleabane). E. Canadense (Horse-weed),

Butterweed); E. bellidifolium (Robin's Plantain); E. Philadelphicum (Common Fleabane); E. annuum (Daisy F., Sweet Scabious); E. strigosum (Daisy Fleabane). Medical properties: Diuretic, astringent, tonic, styptic, diaphoretic, emmenagogue.

Genus Achillea (Yarrow, Sneezewort). *A. millefolium* (Common Yarrow, Milfoil). Medical properties: Astringent, alterative, diuretic, tonic.

Genus Maruta (Mayweed). *M. cotula* (Common Mayweed, Dog-fennel). Medical properties: Bitter, pungent, nervine.

Genus Chrysanthemum. *C. leucanthemum* (Whiteweed, Ox-Eye Daisy). Medical properties: Acrid, tonic, diuretic, antispasmodic; in large doses, emetic.

Genus Helenium (Sneeze-weed). H. autumnale. Medical properties: Tonic, diuretic, diaphoretic, errhine.

Genus Polymnia (Leaf-Cup). P. Canadensis (Leaf-Cup); P. uvedalia (Yellow Leaf-Cup, Bear's-foot). Medical properties: Tonic, antispasmodic.

Genus Silphium (Rosin Plant). S. laciniatum (Rosin-weed, Compass-plant); S. terebinthinaceum (Prairie Dock); S. perfoliatum (Cup-plant). Medical properties: Plant—emetic; root—diuretic, diaphoretic, stimulant, pectoral; gum—stimulant, antispasmodic, pectoral.

Genus Parthenium. P. integrifolium. Medical properties: Aromatic, bitter, stimulant, diuretic, nephritic, lithontryptic.

Genus Coreopsis (Tickseed). C. tripteri—the wildis tripteris (Tall Coreopsis); C. trichosperma (Tickseed, Sunflower). Medical properties: Alterative, expectorant.

Genus Bidens (Bur Marigold). B. frondosa (Common Beggar-ticks); B. connata (Swamp Beggar-ticks); B. beckii (Water Marigold); B. bipinnata (Spanish needles). Medical properties: Expectorant, emmenagogue.

Genus Krigia (Dwarf Dandelion). K. Virginica. Medical properties: Same as Dandelion.

Genus Helianthus (Sunflower). H. giganteus (Wild S.); *H. animus* (Common S.); H. divaricatus (Rough S.); *H. tuberosus* (Jerusalem Artichoke). Medical properties: Diuretic, expectorant, pectoral; the H. tuberosus is edible.

Genus Rudbeckia (Cone Flower). R. laciniata (Common Cone F.). Medical properties: Diuretic, tonic. Also called Thimbleweed.

Genus Echinacea (Hedge Hog, Cone-flower). E. purpurea. Medical properties: Antisyphilitic, depurative, alterative.

Genus Cichorium (Succory, Chickory). C. intybus (Common Chickory). Medical properties: Aperient, deobstruent. bitter.

Genus Hieracium (Hawkweed). H. Scabrum (Rough Hinacium). Medical properties: Bitter, tonic, astringent.

Genus Nabalus (Rattlesnake-root). N. albus (White Lettuce). Medical properties: Bitter, tonic; antivenomous.

Genus Taraxacum (Dandelion). T. Dens-leonis (Common Dandelion). Medical properties: Stomachic, tonic, diuretic, aperient, hepatic.

Genus Lactuca (Lettuce) L. Canadensis (Wild L.); L. sanguinea (Wood or Red L.). Medical properties: Narcotic, anodyne, hypnotic, diuretic, diaphoretic, sedative.

Genus Mulgedium (False or Blue Lettuce). M. acuminatum (Blue L.); M. floridanum (False L.). Medical properties: Leaves considered antivenomous.

Genus Sonchus (Low Thistle). *S. oleraceus* (Common Low Thistle). Medical properties: Bitter, diuretic.

Order Lobeliaceæ (Lobelia Family). 1 genus, 4 species:

Genus Lobelia. L. cardinalis (Cardinal Flower); L. syphilitica (Great Lobelia); L. spicata (Spiked L.); L. inflata (Indian Tobacco, Lobelia). Medical properties: Emetic, expectorant, diaphoretic, diuretic, nervine, antispasmodic, resolvent, anthelmintic, cathartic, antisyphilitic.

Order Campanulaceæ (Campanula Family). 1 genus, 1 species:

Genus Campanula (Bell-flower). C. rotundifolia (Harebell). Medical properties: Emetic, pectoral.

Order Ericaceæ (Heath Family). 7 genera, 11 species:

Genus Guylussacia (Huckleberry). G. resinosa (Common or Black H.). Medical properties: Diuretic, astringent.

Genus Vaccinium (Blueberry). V. macrocarpa (American Cranberry); V. arboreum (Farkleberry); V. Pennsylvanicum (Dwarf Blueberry). Medical properties: Astringent, acid, refrigerant, diuretic, antiscorbutic. Fruit esculent.

Genus Arctostaphylos (Bearberry). A. uva-ursi (Common Bearberry). Medical properties: Diuretic, astringent, tonic, nephritic.

Genus Gaultheria (Aromatic Wintergreen). G. procumbens (Creeping Wintergreen). Medical properties: Stimulant, aromatic, astringent, diuretic, emmenagogue.

Genus Azalea (False Honysuckle). A. nudiflora (Purple Azalea, Pinxter Flower). Medical properties: Astringent.

Genus Pyrola (Shin-leaf). P. rotundifolia (Round-leaved Pyrola); P. elliptica (Shin-leaf). Medical properties: Diuretic, tonic, astringent, antispasmodic.

Genus Chimaphila (Pipsissewa). C. umbellata (Pipsissewa, Prince's Pine). Medical properties: Diuretic, tonic, alterative, astringent, antisyphilitic.

Genus Monotropa (Pine sap). M. uniflora (Indian Pipe, Corpse-plant). Medical properties: Root—tonic, nervine, sedative, antispasmodic.

Order Aquafoliaceæ (Holly Family). 1 genus, 4 species:

Genus Ilex (Holly). I. opaca (American Holly); I. decidua; I. verticillata (Black Alder, Winterberry); I. glabra (Inkberry). Medical properties: Tonic, antiseptic, astringent, emetic.

Order Ebenaceæ (Ebony Family). 1 genus, 1 species:

Genus Diospyros (Date Plum, Persimmon). D. Virginiana (Common Persimmon). Medical properties: Astringent, antiperiodic.

Order Plantaginaceæ (Plantain Family). 1 genus, 4 species:

Genus Plantago (Plantain Ribgrass). P. major (Common Plantain, Waybread); P. cordata (Heart-leaved P.); *P. lanceolata* (Ribgrass, Snake P., English P.); P. Virginira (White P., Dwarf P.). Medical properties: Refrigerant, diuretic, astringent, anodyne, antiemetic, alterative, antisyphilitic.

Order Primulaceæ (Primrose Family). 4 genera, 4 species:

Genus Lysimachia (Loose-strife). L. quadrifolia (Yellow Balm, Crosswort). Medical properties, Astringent, stomachic, antiperiodic, expectorant.

Genus Anagallis (Pimpernel). *A. arvensis* (Poor-man's Weather-glass). Medical properties: Poison, nervine, expectorant, stimulant.

Genus Centunculus (Chaffweed). C. minismus (Bastard Pimpernel). Medical properties: Same as Anagallis.

Genus Samolus (Water Pimpernel, Brookweed). S. valeriandi (Water Pimpernel). Medical properties: Purgative.

Order Bignoniaceæ (Bignonia Family). 3 genera, 3 species:

Genus Tecoma (Trumpet Flower). T. radicans (Trumpet Creeper). Medical properties: Sudorific, antivenomous.

Genus Catalpa (Indian Bean). C. bignonioides (Common Catalpa). Medical properties: Antiasthmatic.

Order Orobranchaceæ (Broom Rape Family). 3 genera, 3 species:

Genus Epiphegus (Buch Drops, Cancer Root). E. Virginianum. Medical properties: Nauseant, bitter, astringent, depurative.

Genus Conopholis (Squaw Root, Cancer Root). C. Americana (Earth Club). Medical properties: Same as preceding genus.

Genus Aphyllon (Naked Broom Rape). A uniflorum (One-flowered Cancer Root). Medical properties: Same as preced ing genus.

Order Scrophulariaceæ (Figwort Family). 8 genera, 16 species:

Genus Verbascum (Mullein). *V. thapsus* (Common Mullein); *V. blattaria* (Moth M.). Medical properties: Demulcent, diuretic, anodyne, antispasmodic, vulnerary; seed—narcotic, purgative.

Genus Veronica (Speedwell). V. Americana (American Brooklime); V. Virginica (Culver's Root or Physic, Leptandra); V. angallis (Water Speedwell); V. Scutellata (Marsh S.); V. peregrina (Purslane S., Neckweed). Medical properties: Antiscorbutic, diuretic, emmenagogue, hepatic, laxative, purgative, tonic, expectorant, alterative.

Genus Linaria (Toad Flax). L. Canadensis (Wild Toad Flax); L. vulgaris (Toad F., Butter and Eggs). Medical properties: Cathartic, diuretic, deobstruent.

Genus Gerardia. G. quercifolia (Smooth False Foxglove, Golden Oak); G. pedicularia (Bushy Gerardia). Medical properties: Sedative, diaphoretic, stimulant, antiseptic, febrifuge.

Genus Gratiola (Hedge Hyssop). G. Virginiana (Water Jessamine; G. Missouriana (?) Medical properties: Carminative (?), acrid, drastic, vermifuge, diuretic, narcotic.

Genus Scrophularia (Figwort). S. nodosa (Healall). Medical properties: Vulnerary, depurative, emmenagogue.

Genus Chelone (Turtle-head, Snake-head, Balmony). C. glabra. Medical properties: Leaves—bitter, tonic, cathartic, anthelmintic, hepatic.

Genus Pedicularis (Lousewort). Canadensis (Wood Betony, Common Pedicularis). Medical properties: Tonic, sedative, astringent.

Order Verbeniceæ (Vervian Family). 1 genus, 4 species.

Genus Verbena (Vervian). V. hastata (Blue Vervian); V. urticifolia (Nettle-leaved or white V.); *V. officinalis* (European V.); V. bractiosa. Medical properties: Emetic, tonic, expectorant, sudorific, rubefacient.

Order Labiate (Mint Family). 19 genera, 27 species:

Genus Tencrium (Germander). T. Canadense (American Germander). Medical properties: Stimulant, tonic, aromatic, bitter, antirheumatic.

Genus Mentha (Mint). M. viridis (Spearmint); *M. piperita* (Peppermint). Medical properties: Aromatic, stimulant, stomachic, carminative, antispasmodic, diuretic; oil, rubefacient.

Genus Lycopus (Water horehound). L. Virginicus (Bulgeweed); L. sinuatus (Paul's Betony, Bitter Bulgeweed); L. Europœus (Bitter Bulgeweed). Medical properties: Tonic, sedative, astringent, pectoral.

Genus Cunila (Ditany). C. Mariana (Common Ditany). Medical properties: Carminative, stimulant, antispasmodic, diaphoretic, tonic, nervine.

Genus Collinsonia (Horsebalm). C. Canadensis (Richweed, Stone-root). Medical properties: Diuretic, antispasmodic, expectorant.

Genus Hedeoma (Mock Pennyroyal). H. pulbyioides (American Pennyroyal). Medical properties: Stimulant diaphoretic, emmenagogue, carminative, sudorific.

Genus Salvia (Sage). S. lyrata (Cancerweed, Wild Sage,

Lyre-leaved S.). Medical properties: Powerfully astringent—used on warts, etc.

Genus Pycanthemum (Mountain Mint, Basil). P. incamum (Wild Basil); P. pilosum; P. linifolium. Medical properties: Tonic, stimulant, diaphoretic, antispasmodic, carminative, sedative.

Genus Satureia (Savoy) *S. hortensis* (Summer Savoy). Medical properties: Aromatic, stimulant, carminative, emmenagogue, aphrodisiac.

Genus Melissa (Balm, Bee-balm, Sweet Herb). *M. officinalis* (Common Balm). Medical properties: Aromatic, diaphoretic, sedative, emmenagogue.

Genus Monarda (Horse Mint, Balm). *M. fistulosa* (Wild Bergamot); M. punctala (Horse Mint). Medical properties: Aromatic, bitter, nervine, stomachic, deobstruent; stimulant, carminative, sudorific, diuretic, emmenagogue, antiemetic.

Genus Blephilia. *B. hirsuta* (Ohio Horse Mint). Medical properties: Aromatic, tonic, stimulant, carminative.

Genus Nepeta (Cat Mint). *N. cataria* (Catnip); *N. glechoma* (Ground Ivy.) Medical properties: Aromatic, carminative, diuretic, antispasmodic, anodyne, emmenagogue, deobstruent, tonic, stimulant.

Genus Physostegia (False Dragon Head). *P. Virginiana.* Medical properties: Aromatic, stimulant; sudorific, diaphoretic, tonic, cerebral sedative.

Genus Prunella. *P. vulgaris* (Self-heal, Heal-all). Medical properties: Astringent.

Genus Scutellana (Skull Cap). S. gabricula (American Skull-cap); S. Cateriflora (Blue Skull-cap). Medical properties: Bitter, antiperiodic, diuretic, tonic, nervine, antispasmodic.

Genus Marrubium (Horehound). *M. vulgare* (Common

H.) Medical properties: Bitter, aromatic, tonic, diaphoretic, expectorant, pectoral, emmenagogue.

Genus Leonurus (Mothroot). *L. cardiaca* (Lion's-tail, Throatwort). Medical properties: Bitter, nervine, emmenagogue.

Genus Stachys (Hedge Nettle). S. palustris (Clown-heal). Medical properties: Nauseant, expectorant, nervine, emmenagogue, vulnerary.

Order Borraginaceæ (Borage Family). 5 genera, 10 species:

Genus Mertensia (Lungwort). M. Virginica (Virginian or Smooth Lungwort). Medical properties: Demulcent, mucilaginous, pectoral.

Genus Onosmodium (False Gromwell). O. Carolinianum; O. Strigosum (Wild Job's-tear); O. Virginianum. Medical properties: Root and seed—diuretic, tonic, lithontryptic.

Genus Lithospermum (Gromwell). *L. arvense* (Corn Gromwell); *L. officinale* (Common G.) Medical properties: Diuretic, lithontryptic.

Genus Cynoglossum (Hound's Tongue). *C. officinale* (Common Hound's T.); C. Virginianum (Wild Comfrey); C. Morisoni (Beggar's Lice). Medical properties: Mucilaginous, tonic, diuretic, astringent, aromatic, anodyne, narcotic.

Genus Heliophytum (Sun-plant). *H. Indicum* (Indian Heliotrope). Medical properties: Bitter, antivenomous, vulnerary, exanthematous.

Order Hydrophyllaceæ (Water-Leaf Family). 1 genus, 1 species:

Genus Hydrophyllum (Water-leaf). H. Virginicum (Burr Flower). Medical properties: Diuretic, astringent.

Order Polemoniaceæ (Polemonium Family). 1 genus, 2 species:

Genus Polemonium (Greek Valerian, Jacob's Ladder). P.

cæruleum (Greek Valerian); P. reptans (American Greek V.) Medical properties: Astringent, vulnerary, alterative, diuretic, diaphoretic, pectoral, expectorant, antivenomous.

Order Convolvulaceæ (Convolvulus Family). 3 genera, 5 species:

Genus Ipomœa (Morning Glory). *I. nil* (Smaller Morning Glory); I. pandurata (Wild Potato Vine, Man of the Earth). Medical property: Cathartic.

Genus Calystigia (Bracted Bindweed). C. sepium (Hedge Bindweed). Medical properties: Diuretic, cathartic.

Genus Cuscuta (Dodder). C. chlorocarpa; C. glomerata (American Dodder). Medical properties: Bitter, tonic, astringent, antiperiodic.

Order Solanaceæ (Nightshade Family). 4 genera, 9 species:

Genus Solanum (Nightshade). S. Carolinense (Horse-nettle); *S. nigrum* (Black or Common Nightshade); *S. Dulcamara* (Bitter Sweet). Medical properties: Narcotic, anodyne, depurative, deobstruent, diaphoretic, discutient; poisonous.

Genus Physalis (Ground Cherry). P. Pennsylvanica; P. pubescens (Common Ground C.); P. viscosa. Medical properties: Diuretic, sedative.

Genus Datura (Thorn-Apple). *D. stramonium* (Stramonium, Jimson or Jamestown Weed); D. tatula. Medical properties: Stimulant, sedative, narcotic, diuretic, diaphoretic, poisonous.

Genus Lycium. *L. vulgare* (Matrimony vine). Medical properties: Astringent, sedative in erysipelas locally.

Order Gentianaceæ (Gentian Family). 5 genera, 11 species:

Genus Sabbatia. S. angularis (American Centaury). Medical properties: Bitter, tonic, hepatic, vermifuge, emmenagogue, febrifuge.

Genus Erythræa (Centaury). E. centaurium (Common European Centaury). Medical properties: Aromatic, bitter, tonic, febrifuge,

Genus Frasera (American Columbo). F. Carolinensis. Medical properties: Bitter, tonic, antiseptic, febrifuge.

Genus Gentiana (Gentian). G. quinqueflora (Five-flowered Gentian); G. crinata (Fringed G.); G. ochrolenca (Sampson Snakeroot, Yellowish White G.); G. Alba (Whitish G.); G. Andrewsii (Closed G.); G. saponaria (Soapwort G.); G. puberula. Medical properties: Bitter, stomachic, tonic, astringent, anthelmintic, cholagogue, febrifuge.

Genus Menyanthes (Buckbean). M. trifoliata. Medical properties: Bitter, tonic, diuretic, emetic, cathartic, anthelmintic.

Order Loganaceæ (Logania Family). 1 genus, 1 species:

Genus Spigelia (Pink-root, Worm-grass). S. Marilandica (Maryland Pink-root). Medical properties: Narcotic, anthelmintic.

Order Apocynaceæ (Dogbane Family). 2 genera, 3 species:

Genus Fœsterania. F. difformis. Medical properties: Astringent, for warts.

Genus Apocynum (Dogbane, Indian Hemp). A. androsæniifolium (Spreading Dogbane, Wandering Milkweed); A. cannabinum (Indian Hemp). Medical properties: Emetic, cathartic, diuretic, sudorific, expectorant, anthelmintic, sternutatory.

Order Asclepiadaceæ (Milkweed Family). 2 genera, 7 species:

Genus Asclepias Milkweed, Silkweed). A. tuberosa (Butterfly weed, Pleurisy-root); A. incarnata (Swamp Milkweed); A. cornuti (Common Milkweed); A. Sullivantii (Smooth M.); A. vertiallata (Whorled M.). Medical properties: Expectorant, diuretic, diaphoretic, carminative, tonic, alterative, emetic, cathartic.

Genus Aceratis (Green Milkweed). A. longifolia; A. viridifolia. Medical properties: Carminative, tonic, diuretic, antispasmodic.

Order Oleaceæ (Olive Family). 1 genus, 3 species:

Genus Fraximus (Ash). F. Americana (White Ash); F. sambucifolia (Black or Water A.); F. quadrangulata (Blue A.). Medical properties: Bark—tonic, diuretic, cathartic, astringent, antiperiodic, discutient, exanthematous; seed—*antiadipose*.

Order Aristolochiaciæ (Birthwort Family). 2 genera, 3 species:

Genus Asarum (Wild Ginger, Asorabacca). A. Canadense (Snakeroot, Southern Wild Ginger). Medical properties: Aromatic, stimulant, diaphoretic, carminative, expectorant.

Genus Aristolochia (Birthwort). A. Serpentaria (Virginia Snakeroot); A. tomentosa. Medical properties: stimulant, tonic, diaphoretic, diuretic, anodyne, febrifuge, exanthematous.

Order Phytolaccaceæ (Pokeweed Family). 1 genus, 1 species:

Genus Phytolacca (Pokeweed). P. decandra (Common P., Scoke, Gayet). Medical properties: Alterative, resolvent, delegent, deobstruent, antisyphilitic, antiscorbutic.

Order Cheuopodiaceæ (Goose-foot Family). 3 genera, 6 species:

Genus Blitum (Blite). B. capitatum (Strawberry blite). Medical properties: laxative.

Genus Chenopodium (Pigweed, Goosefoot). *C. album* (White Goosefoot or Lambsquarter); C. botrys (Jerusalem Oak, Feather Geranium); *C. ambrosoides* (Mexican Tea); *C.*

*anthelminticum* (Wormseed). Medical properties: Antiscorbutic, vermifuge.

Genus Atriplex (Orache). A. potula. Medical properties: Cathartic, drastic.

Order Amarantaceæ (Amaranta Family). 1 genus, 2 species:

Genus Amaranthus (Amaranth). *A. hypochondriacus;* A. albus. Medical properties: Astringent, detergent, antiphlogistic, vulnerary.

Order Polygnaceæ ( Buckwheat Family). 2 genera, 18 species:

Genus Polygonum. *P. Pusicaria* (Lady's-thumb); P. hydropiper (Common Smartweed, Water-pepper); P. acre (Water Smartweed); P. hydropiperoides (Wild Water-pepper); P. amypibium (Water Fersicaria); P.Virginianum; P. arifolium; P. articulatum (Jointweed); P. aviculare (Knotgrass, Goosegrass Doorweed); P. erectum. Medical properties: Astringent, aromatic, purgative, emetic, antiseptic, diuretic, aperient, diaphoretic, tonic, vulnerary.

Genus Rumex (Dock, Sorrel). R. Britanica (Pale Dock); *R. potientia; R. crespus* (Curled D.); *R. obtusifolius* (Bitter D.); *R. acetossella* (Field or Sheep Sorrel). Medical properties: Leaves—acid, refrigerant, diuretic, antiscorbutic, laxative; root—alterative, astringent, depurative, tonic, antiscorbutic.

Order Lauraceæ (Laurel Family). 2 genera, 2 species:

Genus Sassafras. S. officinalis. Medical properties: Bark—aromatic, stimulant, alterative, diuretic, diaphoretic; oil—rubefacient, antirheumatic, stimulant, antiseptic.

Genus Lindera ( Wild Allspice, Firebush). L. benzoin (Spice-bush, Benjamin-bush). Medical properties: Aromatic, anthelmintic, antiperiodic, febrifuge; fruit—spicy.

Order Thymelsaceæ (Mezereum Family). 1 genus, 1 species:

Genus Dirca (Leather-wood, Mosswood). Medical properties: Bark—acrid, rubefacient, vesicant, sudorific, expectorant; twigs—poison.

Order Santalaceæ (Sandal-wood Family). 1 genus, 1 species:

Genus Comandra (Bastard Toad Flax). C. umbellata. Medical properties: Tonic, febrifuge.

Order Loranthaceæ (Mistletoe Family). 1 genus, 1 species:

Genus Phoradendron (False Mistletoe, American M.). P. flaviscens (American Mistletoe). Medical properties: uterine sedative, oxytocic; useful in menorrhagia, irritability of urethra, bladder, etc.; emmenagogue.

Order Sauraceæ (Lizzard's Tail Family). 1 genus, 1 species:

Genus Saururus (Lizzard's Tail). S. cerunus. Medical properties: Emollient, discutient; used for inflamed breasts.

Order Cerataphyllaceæ (Hornwort Family). 1 genus, 1 species:

Genus Ceratophyllum (Hornwort). C. demersum. Medical properties: Demulcent, emollient.

Order Callitrichaceæ (Water Starwort Family). 1 genus, 3 species:

Genus Callitriche (Water Starwort). C. verna; C. heterophylla; C. autumnalis. Medical properties: Diuretic, in dropsy.

Order Euphorbiaceæ (Spurge Family). 3 genera, 16 species:

Genus Euphorbia (Spurge). E. polygonifolia; E. geyeri; E. serpens; E. glyptosperma; E. maculata (Spotted Spurge); E. humistrata; E. hypericifolia (Large Spotted S.); E. corallata

(Blooming S.); *E. helioscopia* (Sun S., Churnstaff); *E. cyparissias* (Cypress S.); E. dentata; E. heterophylia; E. commuta; E. obsutata. Medical properties: Emetic, diaphoretic, expectorant, cathartic, antisyphilitic, astringent, caustic, tonic, narcotic; poisonous.

Genus Acalypha (Three-seeded Mercury). A. Virginiana (Mercury-weed). Medical properties: Expectorant, diuretic.

Genus Ricinus (Palma Christi, Castor-oil Plant); *R. communis.* Medical properties: Laxative, cathartic, emollient.

Order Urticaceæ (Nettle Family). 8 genera, 11 species:

Genus Ulmus (Elm). U. fulva (Slippery Elm); U. Americana (American or White Elm); U. lata (Winged Elm). Medical properties: Astringent, tonic, alterative, diuretic, expectorant; mucilaginous, demulcent, emollient.

Genus Celtis (Hackberry, Nettle-tree). C. occidentalis (Sugar-berry, American Hackberry). Medical properties: Astringent, anodyne, refrigerant.

Genus Morus (Mulberry). M. rubra; M. alba (White Mulberry). Medical properties: Laxative, refrigerant, vermifuge.

Genus Urtica (Nettle). U. dioica (Common Stinging Nettle). Medical properties: Tonic, astringent, diuretic, pectoral.

Genus Pilea (Richweed, Clearweed, Coolweed). P. punula. Medical properties: Astringent, tonic, diuretic.

Genus Parietaria (Pellitory). P. Pennsylvanica (American Pellitory). Medical properties: Diuretic, deobstruent, emmenagogue.

Genus Cannabis (Hemp). *C. sativa* (Common Hemp). Medical properties: Nervine, anodyne, sudorific, antispasmodic.

Genus Humulus (Hops). H. lupulus (Common Hops). Medical properties: Bitter, nervine, tonic, anodyne, hypnotic, diuretic, febrifuge.

Order Platanaceæ (Plane-tree Family). 1 genus, 1 species.

Genus Platanus (Plane-tree). P. occidentalis (American Plane-tree, Sycamore, Buttonwood). Medical properties: Bark—antiscorbutic.

Order Juglandaceæ (Walnut Family). 2 genera, 9 species:

Genus Juglans (Walnut). J. cincrea (Butternut, White Walnut); J. nigra (Black W.). Medical properties: Bark—cathartic, alterative, tonic, anthelmintic, cholagogue, astringent.

Genus Carya (Hickory). C. olivæformis (Pecan); C. alba (Shell-bark or Stag-bark Hickory); C. sulcata (Western Shell-bark); C. tomentosa (Mocku-nut, White-heart Hickory); C. microcarpa (Small Fruited H.); C. porcina (Pig-nut or Brown H.); C. amara (Bitter-nut or Swamp H.). Medical properties: Shell of nut—astringent; Bark—cathartic.

Order Cupulifereæ (Oak Family). 5 genera, 13 species:

Genus Quercus (Oak). Q. alba (White O.); Q. obtusiloba (Post O.); Q. prinus (Swamp Chestnut O,); Q. monticola (Rock Chestnut O.); Q. imbricaria (Laurel or Shingle O.); Q. falcata (Spanish O.); Q. tinctoria (Black O.). Medical properties: Astringent, tonic, antiseptic, bitter, febrifuge.

Genus Castanea (Chestnut). C. Americana (American Chestnut); C. ferruginea (American Beech). Medical properties: Astringent, tonic, febrifuge.

Genus Conylus (Hazel). C. Americana (American Hazelnut or Filbert); C. rostrata (Beaked Hazel). Medical properties: Anthelmintic, diuretic, anodyne.

Genus Ostrya (Hop-Horn Beam, Ironwood). O. Virginica (American Hop-Horn B., etc.). Medical properties: Alterative, tonic, antiperiodic.

Genus Carpinus (Horn Beam, Ironwood). C. Americana

(American Horn Beam, Blue or Water Beech). Medical properties: Same as preceding.

Order Myricaceæ. 1 genus, 1 species:

Genus Comptonia (Solander, Sweet Fern). C. asplenifolia. Medical properties: Astringent, diuretic, tonic, diaphoretic, expectorant, sedative, febrifuge.

Order Betulaceæ (Birch Family). 2 genera, 5 species:

Genus Betula (Birch). B. lenta (Sweet, Black or Cherry Birch); B. alba, var. populifolia (American White B.); B. papyracea (Paper or Canoe B.); B. nigra (River or Red B.). Medical properties: Astringent, bitter, aromatic, excitant, diaphoretic, stimulant; juice—anthelmintic, antiscorbutic; fragrant oil.

Genus Alnus. A. gerrulata (American Tag Alder). Medical properties: Alterative, emetic, astringent.

Order Salicaceæ (Willow Family). 2 genera, 11 species:

Genus Salix (Willow). S. humilis (Prairie Willow); S. cordata (Heart-leaved W.); S. nigra (Black W.); S. eriocephala; *S. fragilis* (Brittle W.); *S. alba* (White W.); *S. Babylonica* (Weeping W.). Medical properties: Bitter, astringent, tonic, antiseptic, deturgent, antiperiodic, antirheumatic.

Genus Populus (Poplar, Aspen). P. tremuloides (American Aspen); P. angulata; P. grandidurtata (Large-toothed A.); P. balsamifera, var. candicans (Balm of Gilead). Medical properties: Stimulant, tonic, diuretic, antiscorbutic, stomachic, antiperiodic, febrifuge.

Order Coniferæ (Pine Family). 7 genera, 9 species:

Genus Pinus (Pine). P. strobus (White Pine). Medical properties: Gum—pectoral, aromatic, bitter, astringent.

Genus Larix (Larch). L. Americana (American Larch or Tamarack). Medical properties: Laxative, tonic, diuretic, alterative.

Genus Taxodium (Bald Cypress). T. distichum (Southern Cyprus). Medical properties: Diuretic, carminative, vulnerary.

Genus Cupressus (Cypress). C. thyoides (White Cedar). Medical properties: Leaves—stimulant, aromatic, diaphoretic, stomachic.

Genus Thuja (Arbor Vitæ). T. occidentalis (American Arbor Vitæ, White Cedar of the North). Medical properties: Stimulant, diaphoretic, anthelmintic, antispasmodic, febrifuge.

Genus Juniperus (Juniper). J. Sabina (Savin); J. Virginiana (Red Cedar); J. Communis (Common Juniper), Medical properties: Bark and leaves—aromatic, stimulant, diuretic, emmenagogue, diaphoretic.

Genus Taxus (Yew). T. baccata, var. Canadensis (American Yew, Ground Hemlock, Dwarf Yew). Medical properties: Nervous sedative.

Order Araceæ (Arum Family). 5 genera, 6 species:

Genus Arisæma or Arum (Indian Turnip). A. tryphyllum (Indian Turnip); A. dracontium (Green Dragon, Dragon-root). Medical properties: Stimulant, expectorant, carminative, diaphoretic.

Genus Peltandra (Arrow Arum). P. Virginica. Medical properties: Stimulant, irritant.

Genus Calla (Water Arum). C. palustris (Wild Water Arum). Medical properties: Root—mucilaginous, stimulant, caustic (?.)

Genus Symplocarpus (Skunk Cabbage). S. fœtidus. Medical properties: Root—expectorant, sudorific, antispasmodic; Fruit and seed—pectoral.

Genus Acorns (Calamus). A. calamus (Sweet Flag). Medical properties: Root—aromatic, carminative, tonic, vulnerary.

Order Typhaceæ (Cat-tail Family). 2 genera, 2 species:

Genus Typha (Cat-tail Flag). T. latifolia (Common Cat-tail or Reed-mace). Medical properties: Root—astringent, emollient, detergent.

Genus Sparganium (Bur-reed). *S. ramosum* or racemosum. Medical properties: Emollient, mildly astringent.

Order Alismaceæ (Water Plantain Family). 2 genera, 2 species:

Genus Alisma (Water Plantain). A. plantago, var. Americana. Medical properties: Leaves—vesicant, diuretic, lithontryptic.

Genus Sagittaria (Arrow Head). S. variabilis (Arrow-weed). Medical properties: Farinaceous, lactagogue.

Order Hydrocharidaceæ (Frog's-bit Family). 1 genus, 1 species:

Genus Valisneria (Eelgrass). V. spiralis (Tape-grass). Medical properties: Plant—refrigerant, demulcent.

Order Orchidaceæ (Orchis Family). 5 genera, 11 species.

Genus Goodyera (Rattlesnake Plantain). G. repens (Net-leaf Plantain); G. pubescens (Adder's-violet). Medical properties: Demulcent, antiscrofulous.

Genus Arethusa. A. bolbosa. Medical properties: Emollient, anodyne.

Genus Corallorhiza (Coral Root). C. multiflora; C. odontorhiza (Crawley-root); Medical properties: Root—diuretic, sudorific, sedative, diaphoretic, febrifuge.

Genus Aplectrum (Putty-root, Adam and Eve). A. hyemale. Medical properties: Root—mucilaginous, pectoral.

Genus Cypripedium (Lady's Slipper, Moccasin Flower). C. arietinum (Ram's-head); C. candidum (Small White Lady's S.); C. parviflorum (Smaller Yellow Lady's S.); C. pubescens (Larger Yellow Lady's S.); C. spectabile (Showy Lady's S.).

Medical properties: Root—tonic, stimulant, diaphoretic, nervine antispasmodic, narcotic, hypnotic.

Order Bromelliaceæ (Pineapple Family). 1 genus, 1 species.

Genus Tillandsia. T. usneoides (Long Moss, Black Moss, Spanish M.). Medical properties: Powder—astringent, in lard for piles.

Order Amaryllidaceæ (Amaryllis Family). 2 genera, 2 species:

Genus Agave (American Aloe). A. Virginica (False Aloe). Medical properties: Root—bitter, carminative.

Genus Hypoxis (Star-grass). H. erecta. Medical properties: Root—detergent, vulnerary, antiperiodic.

Order Hærnodoraceæ (Bloodwort Family). 1 genus, 1 species:

Genus Aleteis (Colic-root, Star-grass). A. forniosa (Unicorn-root). Medical properties: Bitter, tonic, stomachic, uterine sedative.

Order Iridaceæ (Iris Family). 2 genera, 2 species:

Genus Iris (Flower-de-luce). I. versicolor (Larger Blue Flag). Medical properties: Root—alterative, resolvent, sialagogue, laxative, diuretic, antisyphilitic, vermifuge.

Genus Sisyrinchium (Blue-eyed Grass). S. Bermudiana. Medical properties: Root—acrid, cathartic.

Order Dioscoreaceæ Yam Family). 1 genus, 1 species:

Genus Dioscorea. D. villosa (Wild Yam Root). Medical properties: Antispasmodic, diaphoretic; used in bilious and female diseases.

Order Smiliaceæ (Smilax Family). 1 genus, 4 species:

Genus Smilax (Green-brier, Cat-brier, China-brier). S. rotundifolia (Common Green-brier); S. glauca (American Smilax); S. tamnoides (American China-root); S. herbacea (Carrion Flower). Medical properties: Root—depurative, alterative, diuretic, emmenagogue.

Order Liliaceæ (Lily Family). 11 genera, 17 species:

Genus Trillium (Three-leaved Nightshade, Wake-Robin, Birthroot). T. sessile; T. grandiflorum (Large White Trillium); T. erectum (Purple Trillium); T. nivale (Dwarf White T). Medical properties: Root—astringent, tonic, antiseptic, expectorant, diaphoretic, alterative, emmenagogue.

Genus Chamælirium (Devil's Bit.) C. luteum (False Unicorn Root, Blazing Star). Medical properties: Root—alterative, tonic, diuretic, emmenagogue, vermifuge.

Genus Uvularia (Bellwort). U. grandiflora; U. perfoliata. Medical properties: Tonic, demulcent, nervine.

Genus Melanthium (Bunch Flower). M. Virginicum. Medical properties: Insecticide—a sure remedy for itch; antiseptic (?).

Genus Smilacina (False Solomon's Seal). S. racemosa (False Spikenard). Medical properties: Alterative, diuretic, diaphoretic, depurative.

Genus Polygonatium (Solomon's Seal). P. biflorum (Smaller Solomon's Seal); P. gigantum (Great Solomon's Seal). Medical properties: Root—stimulant, cephalic, expectorant, mucilaginous, tonic.

Genus Asparagus. *A. officinalis* (Common Asparagus). Medical properties: Young shoots—diuretic, aperient, deobstruent.

Genus Lilium (Lily). L. Canadense (Wild Yellow Lily). Medical properties: Root—cathartic; maturating, *i. e.*, favoring the formation of pus.

Genus Erythronium (Dog's-tooth Violet). E. Americanum (Yellow Dog's-tooth, Adder's Tongue). Medical properties; Emetic, emollient.

Genus Scilla (Squill). S. Fraseri (Eastern Quamash, Wild Hyacinth). Medical properties: Emollient to inflamed breasts

Genus Allium (Onion, Garlic). A. tricoceum (Wild Leek); A. cernum (Wild Onion); A. Canadense (Wild Garlic). Medical properties: Bulb—Stimulant, diuretic, expectorant, diaphoretic, anthelmintic, emollient; plant—diuretic, stimulant, emollient.

Order Juncaceæ (Rush Family). 1 genus, 2 species:

Genus Juncus (Rush, Bog-rush). J. bufonius (Toad-grass); J. effusus (Bulrush). Medical properties: Plant—cathartic, diuretic.

Order Pontederiaceæ (Pickerel-weed Family). 1 genus, 1 species:

Genus Pontideria (Pickerel-weed). P. cordata. Medical properties: Emollient, astringent.

Order Commelynaceæ (Spiderwort Family). 1 genus 1 species:

Genus Tradescantia (Spiderwort). T. Virginica (Common Spiderwort). Medical properties: Root—demulcent.

Order Xyridaceæ (Yellow-eyed Grass Family) 1 genus, 1 pecies:

Genus Xyris (Yellow-eyed Grass). X. flexuosa. Medical properties: Alterative—in cutaneous diseases.

Order Cyperaceæ (Sedge Family). 1 genus, 1 species:

Genus Eleocharis (Spike Rush). E. polustris. Medical property: Astringent.

Order Gramineæ (Grass Family). 3 genera, 3 species:

Genus Bromus (Brome-grass). B. cilitus. Medical properties: Emetic, anthelmintic, cathartic, diuretic.

Genus Triticum (Wheat). T. repens (Couch, Quitch or Quick Grass). Medical properties: Root—diuretic, aperient.

Genus Phalaris (Canary Seed, Canary Grass). P. canariense. Medical property: Seed—diuretic.

Order Equisetaceæ (Horsetail Family). 1 genus, 4 species:

Genus Equisetum (Horsetail, Scouring Rush). E. arvense (Common Horsetail); E. lævigatum; E. robustum; E. hyemale (Scouring Rush, Shave-grass). Medical properties: Plant—diuretic, astringent.

Order Filices (Fern Family). 7 genera, 12 species:

Genus Polypodium (Polypody). P. incanum (Rock-brake). Medical properties: Root—demulcent, cathartic, anthelmintic.

Genus Adiantum (Maiden Hair). A. pedatum. Medical properties: Plant—pectoral, mucilaginous, expectorant, refrigerant, tonic.

Genus Pteris (Brake). P. aquilina (Common Brake). Medical properties: Root—anthelmintic, in tape worm.

Genus Asplenium (Spleenwort). A. augustifolium; A. trichomanes; A. filix fœmina (Lady Fern). Medical properties: Anthelmintic, astringent.

Genus Aspidium (Shield Fern, Wood F.). A. spinulosum; A. goldianum; A. marginale. Medical property: Anthelmintic.

Genus Osmunda (Flowering Fern). O. regalis (Flowering Fern); O. cinnamomea (Cinnamon Fern). Medical properties: Root—tonic, astringent, mucilaginous, styptic, uterine sedative.

Genus Ophioglossum, O. vulgatum (Adder's-tongue Fern). Medical property: Vulnerary—used in ointments.

Order Lycopodiaceæ (Club-Moss Family). 1 genus, 2 species:

Genus Lycopodium (Club Moss). L. selago (Fir Club Moss); L. lucidulum (Moon-fruit Pine). Medical properties: Emetic, purgative, abortive.

This list comprises about 670 species, or more than one-third of all the plants in the State. There is scarcely a region where there are not plants whose united properties would be sufficient to battle successfully against any ordinary form of disease. We know too little of vegetable materia medica. It is not necessary that search be made for new species, but it is important that we have more definite information concerning the properties of the plants we already know.

Our medical colleges ought to introduce the study of medical botany into their courses of study, even if not more than a dozen lectures were given to it in the term. That would be a beginning, and would lead to closer study and much more perfect knowledge in this important department of a medical education. There is a great tendency at the present time to leave old remedies and prescribe new. It would be better if the old should be more carefully examined and their therapeutic value more accurately determined, that we might rest upon the experience of the past strengthened by that of the present.

With such a list of plants as the physician in Illinois has to select from, there is abundant opportunity to test the principles laid down in the beginning of this article, and to make more exact classifications of our vegetable remedial agents. Not only so, but a broad field is open for the investigation of particular species, in order that we may learn precisely what to expect from a given remedy when it is prescribed at the bedside.

